# A Rare Presentation of Mixed Warm and Cold Autoimmune Hemolytic Anemia

**DOI:** 10.1155/crh/8805562

**Published:** 2026-05-12

**Authors:** Onyekachi Anya, Oluchi Idenyi, Ogbonna Chikere, Joseph J. Shatzel

**Affiliations:** ^1^ Department of Internal Medicine, Legacy Salmon Creek Medical Center, Vancouver, Washington, USA; ^2^ Department of Internal Medicine, WellStar Spalding Regional Hospital, Griffin, Georgia, USA; ^3^ Division of Hematology and Oncology, Oregon Health & Science University, Portland, Oregon, USA, ohsu.edu

## Abstract

Mixed autoimmune hemolytic anemia (mAIHA) is a rare clinical condition where both warm and cold antibodies lead to autoimmune red cell destruction and progressive anemia. Herein, we report a rare case of combined warm and cold agglutinin‐mediated autoimmune hemolytic anemia in a 61‐year‐old male who initially presented with incidental macrocytic anemia during a preoperative evaluation for pterygium surgery. Investigations showed the presence of both warm (IgG) and cold (IgM) autoantibodies, evidenced by a positive direct Coombs test (both anti‐IgG and anti‐C3), and elevated serum IgM levels. Type and cross‐isolate revealed two distinct autoantibodies with different thermal amplitudes. Further workup did not reveal a definitive underlying etiology for the hemolysis. Mycoplasma pneumoniae and Epstein–Barr virus antibody panels were not suggestive of active infection. He was treated with corticosteroids and rituximab, resulting in an improvement in his hemoglobin levels, a reduction in splenomegaly, and ultimately remission of his autoimmune hemolytic anemia. This case emphasizes the need for a thorough workup to identify the cause of anemia, even in the context of seemingly unrelated surgical procedures.

## 1. Background

Autoimmune hemolytic anemia (AIHA) is characterized by the destruction of red blood cells initiated by autoantibodies and/or complement, with macrophages, T‐lymphocytes, and cytokines serving as key amplifiers of the hemolytic process. Warm AIHA (wAIHA) is broadly defined as a condition in which autoantibodies, predominantly of the IgG subclass, display optimal binding to RBC antigens at body temperature (37°C) [1]. In cold AIHA (cAIHA), autoantibodies are usually of the IgM subclass and bind to RBC antigens most efficiently at lower temperatures (4°C), typically in the range of 0°C–4°C [2].

wAIHA is the most common form of AIHA. In wAIHA, IgG autoantibodies are generally directed against Rh complex antigens on the RBC membrane, causing extravascular hemolysis (principally in the spleen) [3]. In contrast, cAIHA is mediated more often by IgM antibodies with specificity for I/i antigens. cAIHA is dominated by extravascular hemolysis but can rarely include intravascular hemolysis depending on the extent of terminal complement activation. This occurs if complement activation proceeds beyond C3, leading to the formation of C5 convertase and, eventually, the membrane attack complex. This is very important when choosing a therapeutic intervention [4–6].

While the two main categories of AIHA are warm (wAIHA) and cold (cAIHA), in exceedingly rare circumstances, some patients can present with both warm and cold autoantibodies, resulting in what is termed mixed AIHA [2]. Mixed AIHA presents diagnostic and therapeutic challenges due to the interrelationship between IgG and IgM hemolysis [7]. The underlying mechanisms driving mixed AIHA remain poorly defined, further complicating diagnosis. As a result, a thorough diagnostic evaluation is often necessary, incorporating serologic testing, flow cytometry, and assessment for systemic conditions such as lymphoproliferative disorders, infections, or other autoimmune diseases. Management requires individualized therapeutic strategies that address both warm and cold autoantibodies, with careful consideration of ongoing hemolysis and the potential toxicities of treatment [8].

In the following case, a 61‐year‐old male with macrocytic anemia and leukopenia underwent a preoperative evaluation to ultimately find that he had a rare case of combined warm and cold agglutinin‐mediated AIHA of undetermined origin. We also describe the clinical presentation, diagnostic findings, and initial management of this rare condition.

## 2. Case Presentation

### 2.1. Patient History

A 61‐year‐old male with a past medical history of autoimmune hepatitis (which spontaneously resolved without treatment) was referred for preoperative evaluation before left eye pterygium surgery.

Initial laboratory investigations on the day of presentation revealed a hemoglobin level of 9.1 g/dL, a mean corpuscular volume (MCV) of 110.7, and mild leukopenia with a white blood cell (WBC) count of 3.0 × 10^9/L. The ANC was 2.20. The reticulocyte count was elevated, and the C‐reactive protein (CRP) was within the normal range. On peripheral smear, normal neutrophil and platelet count morphology was noted. Prominent red cell agglutination was seen, limiting optimal morphologic assessment of both RBCs and WBCs. No circulating blasts were noted. Full laboratory data at the time of diagnosis are outlined in Table 1.

**TABLE 1:** Hematologic and immunohematologic laboratory trends.

Test	Day 1	Day 2	Day 3	Day 10	Reference range
Hemoglobin (g/dL)	9.1	7.0	7.5	6.8	13.5–17.5
WBC (× 10^9/L)	3.0	2.8	3.13	2.9	4.0–11.0
Neutrophils, absolute	1.9	1.7	1.90	1.8	1.5–7.8 10∗3/μL
Lymphocytes, abs	0.8	0.9	0.94	0.9	0.9–3.9 10∗3/μL
Monocytes, absolute	0.2	0.5	0.18	0.2	0.2–1.0 10∗3/μL
Eosinophils, absolute	0.1	0.1	0.06	0.0	0.0–0.5 10∗3/μL
Basophils, absolute	< 0.0	< 0.0	0.03	0.0	0.0–0.2 10∗3/μL
MCV (fL)	110.7	117	112.3	119.2	80–100
Retic. %	7.36	—	—	0.9	0.5–2.5
Platelets (× 10^9/L)	248	217	215	215	150–450
Haptoglobin (mg/dL)	—	—	< 3	—	30–200
Coombs test	—	—	Positive	—	Negative
Anti‐C3	—	—	Positive	—	Negative
Anti‐IgG	—	—	Positive	—	Negative
Cold agglutinin	Strong +	—	1:2	—	Negative
IgM (mg/dL)	—	—	247	—	40–230

*Note:* Hb–hemoglobin. Retic–reticulocyte percentage. DAT–direct antiglobulin (Coombs) test. IgG/IgM–immunoglobulin G/M. C3–complement component 3. Abs–absolute count. fL–femtoliters. mg/dL–milligrams per deciliter. × 10^9^/L–10^9^ cells per liter.

Abbreviations: MCV, mean corpuscular volume; WBC, white blood cell count.

Follow‐up laboratory studies demonstrated progressive anemia, with hemoglobin declining from 9.1 g/dL on Day 1 to 6.8 g/dL by Day 10. The anemia was progressively macrocytic, reflected by a persistently elevated MCV of 119.2 fL, and was accompanied by an initial reticulocytosis of 7.36% and a markedly reduced haptoglobin level (< 3 mg/dL), consistent with a compensatory erythropoietic response to hemolysis.

Further serologic evaluation revealed an elevated serum immunoglobulin M (IgM) level of 247 mg/dL and a positive direct antiglobulin test demonstrating both anti‐C3 and anti‐IgG components. The reported cold agglutinin titer was 1:2; however, this appeared discordant with the marked red cell agglutination observed on peripheral smear and the elevated IgM level, raising concern for potential underestimation. As testing was performed at an external reference laboratory, confirmation of specimen handling conditions and repeat titration were not feasible. Antibody screening was positive, with identification demonstrating an E‐negative and K‐negative red cell phenotype and the presence of both warm and cold autoantibodies. Tests for mononucleosis, hepatitis B, and hepatitis C were negative. Epstein–Barr virus (EBV) qualitative PCR was positive; however, the EBV antibody panel did not indicate active infection, and viral quantification was not performed. Serum flow cytometry showed no monoclonal B‐cell or aberrant T‐cell populations and no circulating blasts. These findings confirmed mixed AIHA, with both IgG (warm) and IgM (cold) autoantibodies mediating hemolysis.

### 2.2. Hospital Admission and Treatment

The patient was admitted 10 days after his initial presentation with a hemoglobin of 6.8 g/dL. Prednisone was initiated at 1 mg/kg, and rituximab was started at 375 mg/m^2^ IV weekly for four doses. Although transfusion was initially considered, it was deferred because serologic testing was complicated by autoantibody interference, limiting the ability to exclude clinically significant alloantibodies and necessitating the consideration of extended antigen–matched red blood cell units. His hemoglobin subsequently improved with immunosuppressive therapy alone, rising to 7.5 g/dL without transfusion.

CT scan of the chest, abdomen, and pelvis revealed splenomegaly (16.1 cm) and mildly enlarged right pelvic sidewall lymphadenopathy (12 mm). The patient’s hemoglobin fluctuated between 6.8 and 7.7 g/dL during the admission, and he remained asymptomatic and hemodynamically stable. He was discharged home with instructions to avoid cold temperatures, a plan for weekly laboratory monitoring (CBC/diff, CMP, LDH, and reticulocytes), and a repeat CT scan in approximately 2 months if hemolysis remained controlled.

### 2.3. Follow‐Up

Follow‐up laboratory testing 5 weeks later demonstrated an improved hemoglobin of 12.6 g/dL. Repeat CT imaging showed stable lymphadenopathy but revealed interval improvement in splenomegaly, with the spleen measuring 11.8 cm compared to 16.1 cm previously.

## 3. Discussion

This case highlights a rare instance of AIHA mediated by both warm and cold agglutinins. The simultaneous presence of IgG and C3 positivity on the direct Coombs test, along with a positive cold agglutinin test, and eventual antibody identification confirmed the coexistence of warm and cold autoantibodies.

Given the absence of underlying autoimmune disease, prior transfusions, acute infection, or evidence of a lymphoproliferative disorder, the patient’s mixed warm and cold AIHA was ultimately deemed to be idiopathic.

The management of mixed AIHA is often challenging because responses to therapies used for wAIHA (e.g., corticosteroids and splenectomy) or cAIHA (e.g., cold avoidance or B‐cell–directed therapies) can be variable. In this case, the patient was initially treated with prednisone, which resulted in a modest improvement in hemoglobin levels. Subsequently, rituximab, a B‐cell–depleting agent effective in many cases of cAIHA and in some instances of wAIHA, was administered, with a good long‐term response. Splenomegaly noted on CT imaging is a common finding in AIHA, reflecting increased splenic sequestration and destruction of red blood cells, and in this case, resolved with remission of hemolysis. Although our patient did not require complement‐directed therapy, it is important to highlight options for severe or refractory hemolysis when the cold component is a major driver, such as in mixed AIHA with prominent cold agglutinin activity.

In autoimmune hemolysis (AIHA) with significant cold‐agglutinin or complement‐mediated hemolysis, targeted inhibition of the complement cascade has emerged as an important therapeutic strategy. Cold agglutinin disease (CAD) is driven by IgM autoantibodies that activate the classical pathway, resulting in C3b deposition and extravascular hemolysis, with occasional intravascular hemolysis via the membrane attack complex formation.

Sutimlimab, a humanized monoclonal antibody targeting complement C1s, inhibits the classical pathway, preventing complement‐mediated RBC destruction. In the Phase 3 CADENZA and CARDINAL trials, sutimlimab rapidly and sustainably increased hemoglobin, reduced markers of hemolysis, and decreased transfusion requirements in adults with CAD [4, 9]. The therapy is generally well tolerated, though it requires ongoing infusions and does not suppress autoantibody production.

Eculizumab, a terminal complement (C5) inhibitor, has also demonstrated efficacy in CAD and cAIHA, primarily reducing intravascular hemolysis and transfusion requirements, though hemoglobin responses may be less pronounced than with proximal blockade [10, 11]. Its use is typically reserved for severe or refractory hemolysis.

In mixed AIHA, where both warm IgG and cold IgM autoantibodies contribute to hemolysis, complement inhibition may be considered early in cases where the cold component drives significant hemolysis. While most data are derived from pure CAD, mechanistic rationale and cases support its use in mixed disease [12].

We conducted a literature review of published case reports and interventional clinical studies from 2011 to 2025 examining wAIHA, CAD, and mixed warm–cold agglutination. Across reports, patients commonly presented with severe, transfusion‐requiring anemia. First‐line therapy predominantly included corticosteroids, with variable responses in mixed and cold phenotypes. Rituximab was the most frequently used second‐line agent and was associated with durable responses in refractory cases. Complement‐directed therapies, including sutimlimab and eculizumab, demonstrated rapid hematologic improvement in cold agglutination, while emerging therapies such as daratumumab showed promising efficacy in refractory wAIHA and CAD. Overall, treatment response varied by AIHA subtype and underlying etiology, highlighting the need for phenotype‐directed therapy. See Table 2.

**TABLE 2:** Reported patient characteristics, severity, treatment, and outcomes in warm and cold agglutination from published literature.

Study/journal (year)	Age (yrs)	Sex	AIHA type	Associated condition	Severity at presentation	Treatment(s) used	Time to response/outcome
Reddy et al. (2011). PubMed [13].	12	Female	Mixed warm and cold	Unknown	Severe anemia (Hb 3 g/dL)	IV corticosteroid	Responded well to 2 transfusions and corticosteroid
Gupta et al., Journal of Medical Case Reports (2011) [14]	62	Male	Mixed warm and cold	Unknown	Severe anemia (Hb 4.5 g/dL), transfusion‐dependent	IV corticosteroids ⟶ plasmapheresis (no response) ⟶ rituximab 375 mg/m2 every week (4 weekly doses)	Complete remission after rituximab; sustained remission at 2 years
Anderson et al. BMJ Case Reports (2020) [15]	31	Male	Warm and cold	SLE	Hb (from 8.1 to 6.3 gm/dL)	2 days of IVIG overlapped with 3 days of methylprednisolone 1 g, followed by prednisone 1 mg/kg (100 mg) daily	Complete remission.
Imataki et al., (2020) [16]	66	Male	Mixed warm and cold	Idiopathic. Had a transfusion with prior stem cell transplant	Severe anemia (Hb 4.1 g/dL)	Prednisone 1 mg/kg. Duration not specified	Initial response to prednisolone, but the disease progressed, and the patient died of acute cardiopulmonary arrest
Charles Smith et al. Case Report in Hematology 2023 [17].	83	Female	Mixed cold and warm	Idiopathic	Severe anemia (Hb 59 g/L)	Prednisone 1 mg/kg; >> rituximab 375 mg/m2	No response to initial Prednisone. Responded well to rituximab with hemoglobin stabilization
Hanna and Carção (2023) [18].	6	Female	Mixed cold and warm	URTI	Severe anemia (Hb 2.1g/dL)	Prednisone 4 mg/kg/day for 4 days >> 2 mg/kg/day for 11 days >> 1 mg/kg/day for 7 days and 0.5 mg/kg/day for 4 weeks >> mycophenolate mofetil 240 mg twice daily	Partial response to initial prednisone Improved response mycophenolate mofetil
Rajput et al. APIK Journal of Internal Medicine (2025) [20].	14	Female	Mixed warm and cold	SLE	Severe anemia (5.4 g/dL)	Oral prednisolone (40 mg) daily for 1 month and tapered after 1 month of treatment, and no blood transfusion was given.	Responded well to prednisolone
Zhang et al., J Med Case Rep (2025) [21]	37	Female	Warm AIHA	(DLBCL‐associated)	Hb 55 g/L	IVIG (20 g/day for 7 days); rituximab 375 mg/m2 on Day 1; R‐CHOP chemotherapy: cyclophosphamide 750 mg/m2 on Day 2, doxorubicin 50 mg/m2 on Day 2, and prednisone 100 mg on Days 2–6, administered every 3 weeks for two cycles.	Gradual response with combination immunochemotherapy
Zhang et al., Journal of Medical Case Reports (2025) [21]	29	Male	Cold AIHA	(DLBCL‐associated)	Hb 58 g/L	Initial oral prednisone acetate at a dose of 15 mg (three times daily) and IV rituximab 100 mg once weekly for four doses. oral prednisone acetate at a dose of 15 mg (three times daily)	Gradual response with combination immunochemotherapy
Röth et al. NEJM (2021) [4].	Median 71	Mixed	Cold agglutinin disease (CAD)	None	Median Hb 8.7 Range (4.9–11.1)	Sutimlimab	Sutimlimab treatment normalized bilirubin levels and increased mean hemoglobin levels, and most patients did not receive a transfusion from week 5 through week 26.
Jalink et al. Blood Advances (2024) [5]	Median 60	Mixed	Refractory wAIHA (n = 12)	Mixed. 5 patients with Evans syndrome and 3 patients with secondary wAIHA (1 with myasthenia gravis; 2 with SLE).	Hb 8.5 g/dL. (range, 4–11.2).	Daratumumab monotherapy	Median time to response 2 weeks (range, 2–8)
Jalink et al. Blood Advances (2024) [5]	Median 66	Mixed	Refractory CAD (n = 7)	Mixed. 2 were diagnosed with lymphoplasmacytic lymphoma and 5 with primary CAD	Hb 8.6 g/dL	Daratumumab monotherapy	Median time to response in 3 weeks
Fujisawa et al. CEN Case report (2024) [6]	73	Male	Severe cold AIHA	Unknown. Patient undergoing HD	Hb 7.1 g/dL	Rituximab (500 mg/dose) weekly (four times in total) >>> sutimlimab (6.5 g/dose). Unknown number of doses given.	After initiating sutimlimab treatment, Hb levels increased despite a decrease in temperature. Six months after the initiation of sutimlimab, the Hb levels in the patient remained stable
Turudić et al. Frontier; PubMed; 2023 [12]	5	Male	Mixed AIHA, transfusion‐dependent	EBV and CMV	Hb 4.4 and HCT 14.9 on presentation	IV methylprednisolone (4 mg/kg body weight) for the first 72 h, followed by 2 mg/kg body weight for 21 consecutive days and very slow steroid reduction until plasmapheresis; rituximab (375 mg/m2); PLEX (five cycles in total, with side effects such as an increased tendency to clotting) and IVIg (1 g/kg/5 days) with vitamins B9 and B12	This combination of medications significantly improved RBC count and platelet levels. Response over 4–6 weeks
Garg et al. BJHeam (2021) [22]	58	Male	Warm agglutination	Idiopathic	Severe Hb (64 g/L)	Rituximab was initiated as a weekly 375 mg/m2 infusion (total dose unknown), and mycophenolate mofetil (MMF) 1 g twice daily, and intravenous Ig 1 g/kg/day. (total doses unknown). Eculizumab single dose 600 mg. No added dosage noted.	Hemolysis stabilized with the addition of eculizumab

*Note:* Hb–hemoglobin. HCT–hematocrit. IV–intravenous. g/dL–grams per deciliter. mg/m^2^–milligrams per square meter. g–grams. IVIG–immunoglobulin. mg/kg–milligram per kilogram. g/L–grams per liter. wAIHA–warm antibody hemolytic anemia. CMV–cytomegalovirus. R‐CHOP–rituximab (or “R”), cyclophosphamide (or “C”), doxorubicin (hydroxydaunorubicin, or “H”), vincristine (oncovin, or “O”), and prednisone (or “P”). PLEX–plasma exchange.

Abbreviations: AIHA, autoimmune hemolytic anemia; CAD, cold agglutinin disease; DLBCL, diffuse large B‐cell lymphoma; EBV, Epstein–Barr Virus; SLE, systemic lupus erythematosus; URTI, upper respiratory tract infection.

## 4. Conclusion

This case demonstrates a rare presentation of combined warm (IgG‐mediated) and cold (IgM‐mediated) AIHA in a 61‐year‐old male. The diagnostic evaluation confirmed the simultaneous presence of both types of autoantibodies, which contributed to significant hemolysis. Initial management with corticosteroids and rituximab resulted in hematologic improvement, with close monitoring of laboratory and imaging findings. While mixed AIHA is often idiopathic, this case highlights the complexity of diagnosis and management, underscoring the importance of individualized treatment strategies based on comprehensive laboratory and clinical assessments.

## Funding

No funding was received for this manuscript.

## Conflicts of Interest

The authors declare no conflicts of interest.
